# Efficacy and safety of Buzhong Yiqi decoction combined with surgery for rectal prolapse: A protocol for systematic review and meta analysis

**DOI:** 10.1097/MD.0000000000022732

**Published:** 2020-10-09

**Authors:** Yanpeng Xie, Yihua Fan, Chen Yang, Renhong Wan, Xiaoen Cheng, Xiangdong Yang, Yuanzhang Hu, Changyou Deng

**Affiliations:** a Chengdu Anorectal Hospital, Chengdu, Sichuan Province; b First Teaching Hospital of Tianjin University of Traditional Chinese Medicine; c National Clinical Research Center for Chinese Medicine Acupuncture and Moxibustion; d Tianjin University of Traditional Chinese Medicine; e School of Medical Information Engineering, Chengdu University of Traditional Chinese Medicine, Chengdu; f Longwang Town Central Hospital, Guangyuan, Sichuan Province, China.

**Keywords:** Buzhong Yiqi decoction, meta-analysis, protocol, rectal prolapse, surgery, systematic review

## Abstract

**Background:**

It is extremely easy for rectal prolapse to relapse with surgery alone. Clinical practice indicates that Buzhong Yiqi decoction combined with surgery has certain therapeutic advantages, while there is a lack of evidence-based medicine support. This study aimed to systematically investigate the efficacy and safety of Buzhong Yiqi decoction combined with surgery in the treatment of rectal prolapse.

**Methods:**

The English databases (PubMed, Embase, Web of Science, the Cochrane Library) and Chinese databases (China National Knowledge Infrastructure [CNKI], Wanfang, China Science and Technology Journal Database [VIP], China Biology Medicine disc) were searched by computer. In addition, Baidu Scholar and Google Scholar were searched manually. A randomized controlled clinical study of Buzhong Yiqi decoction combined with surgery in the treatment of rectal prolapse was performed from the establishment of databases to September 2020. Two investigators independently conducted data extraction and assessed the literature quality of the included studies. The Revman5.3 software was used for meta-analysis of the included literature.

**Results:**

The efficacy and safety of Buzhong Yiqi decoction combined with surgery in the treatment of rectal prolapse were evaluated in terms of efficiency, symptom score, recurrence rate, adverse reaction rate, and so on.

**Conclusions:**

Thisstudy provides reliable evidence-based support for the clinical application of Buzhong Yiqi decoction combined with surgery in the treatment of rectal prolapse.

**OSF Registration number:**

DOI: 10.17605/OSF.IO/K3PJX.

## 1 Introduction

Rectal prolapse is a chronic disease in which the anal canal, rectal mucosa, the full-thickness of the rectum, and part of the sigmoid colon are displaced downward and prolapsed outside the anus. It can be divided into external rectal prolapse and internal rectal prolapse or rectal intussusception.^[1]^ The incidence of rectal prolapse is not high and is estimated to occur in less than 0.5% of the population.^[2]^ Rectal prolapse occurs at the extremes of age,^[3]^ of which the frequency is higher in children, the elderly, and females. The age of incidence for children is generally before 4-year-old,^[4]^ for the elderly is generally after 50-year-old, the proportion of females is higher than that of men (ratio 9:1),^[1]^ and the higher incidence occurs with the older age.^[5]^ The clinical manifestations include pain, bleeding, and fecal incontinence.^[1,6]^ Long-term rectal prolapse can cause damage to the pudendal nerve, which leads to anal incontinence, ulcers, bleeding, necrosis, and so on. Currently, surgery is the main treatment for rectal prolapse, while there are various kinds of surgical methods with quite different treatment effects. Some surgeries require extensive dissection, which causes severe damage to tissues, and many postoperative complications and sequelae will occur. Improper selection of surgical methods could lead to the recurrence of prolapse or disorder in defecation.^[7–9]^ New therapeutic regimes are needed because they cannot solve the patient's pain with surgery alone.

Rectal prolapse belongs to the category of “rectal prolapse (Tuo Gang)” in traditional Chinese medicine, the pathological mechanism of which includes the deficiency of spleen qi, sinking of middle-qi, and relaxation change of the mucous membrane caused by the weakness of large intestine transmission. Therefore, the treatment should be mainly on invigorating the spleen stomach and replenishing qi. It has been proved clinically that the herbs of benefiting qi can improve muscle tension relaxation.^[10]^ Buzhong Yiqi Decoction is a well-known prescription for treating the sinking of middle-qi and has long been used for the treatment of gastrointestinal diseases.^[11]^ Recently, it has been widely used for the treatment of rectal prolapse.^[12–14]^

At present, there are multiple randomized controlled studies showing the results that the treatment of Buzhong Yiqi Decoction combined with surgery for rectal prolapse could strengthen the treatment effect and reduce the recurrence rate.^[15–17]^ However, there were differences in research plans and treatment effects among various clinical trials, resulting in varied research results and restriction of the promotion of this method to a certain extent. Therefore, this study aimed to systematically assess the efficacy and safety of Buzhong Yiqi decoction combined with surgery in the treatment of rectal prolapse and provide a reliable reference for the clinical application of Buzhong Yiqi decoction in the treatment of rectal prolapse.

## 2 Methods

### 2.1 Protocol register

This protocol of systematic review and meta-analysis has been drafted under the guidance of the preferred reporting items for systematic reviews and meta-analysis protocols (PRISMA-P). Moreover, it has been registered on the open science framework (OSF) on September 4, 2020. (registration number: DOI 10.17605 / OSF.IO / K3PJX).

### 2.2 Ethics

Since this is a protocol with no patient recruitment and personal information collection, approval by the ethics committee is not required.

### 2.3 Eligibility criteria

#### 2.3.1 Types of studies

We will collect all available randomized controlled trials (RCTs) on Buzhong Yiqi Decoction combined with surgery in the treatment of rectal prolapse, regardless of blinding, publication status, and region, but language will be restricted to Chinese and English.

#### 2.3.2 Patients

The patients were definitively diagnosed with rectal prolapse, with no limitation of nationality, race, age, sex, course of disease, etc.

#### 2.3.3 Intervention

Patients in the treatment group received Buzhong Yiqi decoction combined with surgery, while patients in the control group underwent surgery alone. There were no restrictions on the dose, frequency, and treatment course of Buzhong Yiqi decoction as well as the surgical methods.

#### 2.3.4 Outcome indicators

(1) Primary outcome: the overall effective rate (the overall effective rate = the number of cured + the number of effective)/ the total number × 100%. (the evaluation standard of rectal prolapse in the “Guidelines for the Diagnosis and Treatment of Common Diseases in the Anorectal Department of Traditional Chinese Medicine” issued by the China Association of Chinese Medicine in 2012.^[18]^ Cure: The clinical symptoms disappeared, the rectal mucosa was smooth under anoscopy, and no relaxed or prolapsed mucosa was seen. Effective: The clinical symptoms were basically disappeared or relieved, and the rectal mucosa was smooth under anoscopy, and local relaxed or prolapsed mucosa was still found. Ineffective: There was no improvement in clinical symptoms and relaxed or prolapsed mucosa were still found under the anoscopy.)
(2) Secondary outcomes:
  - symptom scores;^[19]^
  - recurrence rate; and adverse reaction rate.

### 2.4 Exclusion criteria

(1) Repeatedly published papers
(2) The published papers were abstracts or the data were incomplete and the papers with complete data were not available after contacting the author.
(3) Papers with obvious data errors;
(4) Papers assessed as high risk of bias by randomization or concealed distribution.^[20]^
(5) Papers whose intervention was combined with other traditional Chinese medicine therapies, such as acupuncture treatment and auricular point plaster therapy.
(6) Papers with no relevant outcome indicators.

### 2.5 Retrieval strategies

Take “Buzhong Yiqi Decoction (bu zhong yi qi tang),” “Rectal prolapse (zhi chang tuo chui),” “Rectal mucosal prolapse (zhi chang nian mo tuo chui),” “prolapse of rectum (tuo gang)” as the Chinese search terms and search in Chinese databases, including China National Knowledge Infrastructure (CNKI), Wanfang, China Science and Technology Journal Database (VIP), China Biology Medicine disc; Take “Buzhong Yiqi,” “Anus Prolapses,” “Prolapse,” “Rectal,” “Rectal Prolapses” as the English terms and search terms and search in English databases, including PubMed, EMBASE, Web of Science, the Cochrane Library. In addition, Baidu Scholar and Google Scholar were searched manually. The retrieval time was from the establishment of databases to September 2020, collecting all domestic and international literatures about Buzhong Yiqi Decoction combined with surgery on the treatment of rectal prolapse. Taking PubMed as an example, the retrieval strategy is shown in Table 1.

**Table 1:**
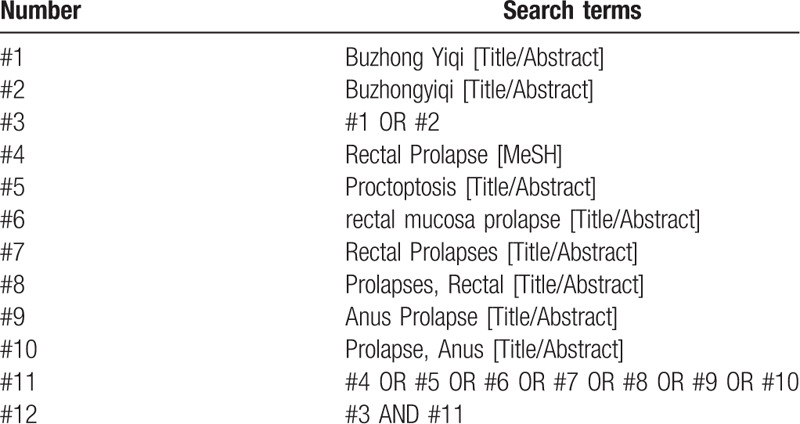
Retrieval strategy of PubMed.

### 2.6 Data filtering and extraction

**Figure 1:**
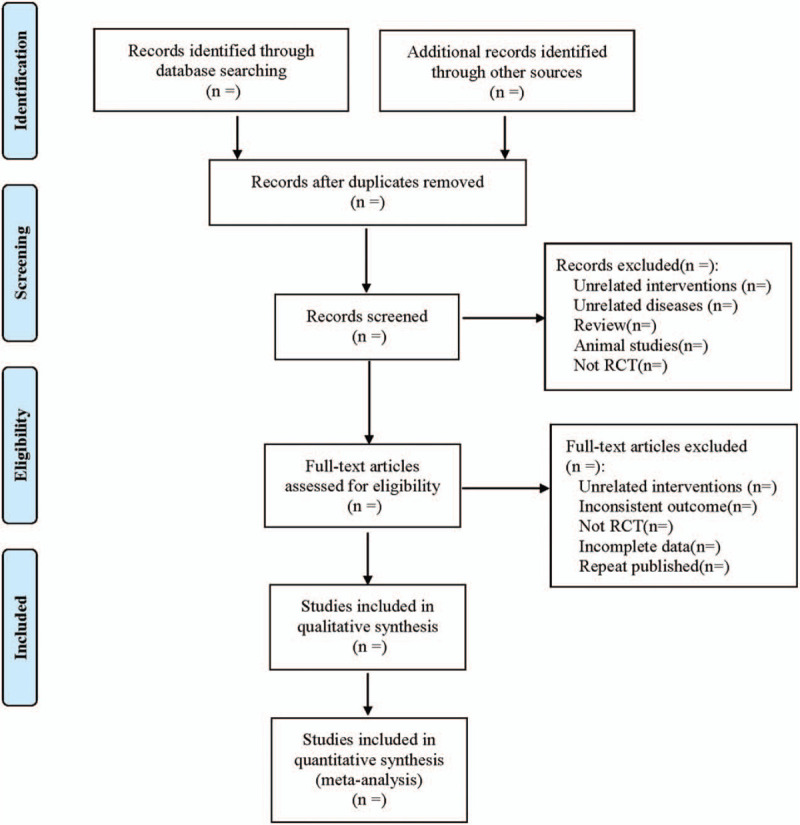
The process of literature filtering.

Referring to the method of research selection in version 5.0 of the Cochrane Collaboration Network System Evaluator Manual, according to the preferred reporting items for systematic reviews and meta-analysis (PRISMA) flow chart, the 2 researchers used the EndNote X9 document management software to independently screen and check the literature according to the above inclusion and exclusion criteria, and check each other, if there were different opinions, negotiate with a third party to resolve the differences. At the same time, Excel 2013 was used to extract relevant information, including:

(1) Clinical research (title, first author, year of publication, sample size, sex ratio, average age, average course of disease, prolapse type);
(2) intervention (the ingredients, doses, frequency, treatment course of Buzhong Yiqi decoction in the treatment group; specific operative schemes in the treatment and control groups);
(3) Evaluation elements of various risk biases in RCTs.
(4) Outcome indicators. The process of literature filtering is shown in Figure 1.

### 2.7 Literature quality assessment

Use the Cochrane collaboration's tool for assessing risk of bias to assess the risk of bias assessment of included studies. According to the performance of the included literature in the above evaluation items, 2 researchers will give judgments like low risk, unclear or high-risk judgments one by one, and cross-check after completion, respectively. In case of any disagreement, a discussion will be carried out. If no agreement can be reached between the 2, a discussion will be made with the researchers in the third party.

### 2.8 Statistical analysis

#### 2.8.1 Data analysis and processing

The RevMan 5.3 software provided by the Cochrane Collaboration will be used for statistical analysis. Relative risk was selected as the statistic for the dichotomous variable. For continuous variables, the weighted mean difference is selected when the tools and units of measurement indicators are the same, the standardized mean difference is selected with different tools or units of measurement, and all the above are represented by the effect value and 95% confidence interval. Heterogeneity test: *The Q test is used to qualitatively determine inter-study heterogeneity. If P* ≥ .1, there is no inter-study heterogeneity; if *P* < .1, it indicates inter-study heterogeneity. At the same time, *the I*^*2*^ value was used to quantitatively evaluate the inter-study heterogeneity. If *I*^*2*^ ≤ 50%, the heterogeneity is considered to be good, and the fixed-effect model is adopted. If *I*^*2*^ > 50%, it is considered to be significant heterogeneity, the source of heterogeneity will be explored through subgroup analysis or sensitivity analysis. If there is no obvious clinical or methodological heterogeneity, it will be considered as statistical heterogeneity, and the random-effect model will be used for analysis. Descriptive analysis will be used if there is significant clinical heterogeneity between the 2 groups, and subgroup analysis is not available.

#### 2.8.2 Dealing with missing data

If there are missing data in the article, contact the author via email for additional information. If the author cannot be contacted, or the author has lost relevant data, descriptive analysis will be conducted instead of meta-analysis.

#### 2.8.3 Subgroup analysis

According to the type of rectal prolapse, it could be divided into complete rectal prolapse and rectal mucosal prolapse for subgroup analysis, subgroup analysis according to the course of treatment, and subgroup analysis was performed according to different operative schemes.

#### 2.8.4 Sensitivity analysis

In order to test the stability of meta-analysis results of indicators, a one-by-one elimination method will be adopted for sensitivity analysis.

#### 2.8.5 Assessment of reporting biases

Funnel plots were used to assess publication bias if no fewer than 10 studies were included in the outcome measure. Moreover, Egger and Begg tests were used to evaluate potential publication bias.

#### 2.8.6 Evidence quality evaluation

Grading of Recommendations Assessment, Development, and Evaluation (GRADE) will be used to assess the quality of evidence. It contains 5 domains (bias risk, consistency, directness, precision, and publication bias). The quality of evidence will be rated as high, moderate, low, and very low.

## 3 Discussion

Studies have indicated that the pathologic of rectal prolapse includes the presence of an abnormally deep pouch of Douglas, relaxation of the pelvic floor muscles and anal canal muscle, weakness of the internal and external sphincter, the lack of normal fixation of the rectum, with a mobile mesorectum and lax lateral ligaments.^[21]^ Traditional medicine believes that the pathological mechanism of rectal prolapse is the sinking of qi caused by the insufficiency of middle qi and nonconsolidation due to deficiency of qi.^[22]^ The treatment should be mainly on invigorating the spleen stomach and replenishing qi. Current regular treatments are aimed at controlling prolapse, restoring continence, and preventing constipation or impaired evacuation^[23]^ via surgery, but the clinical effect is not satisfactory and easy to relapse. Buzhong Yiqi Decoction is a famous prescription in ancient China that has been used for more than 700 years in China and has been widely used in the sinking of qi due to qi deficiency.^[24,25]^ The composition of Buzhong Yiqi Decoction is Radix Astragali (Zhi Huang Qi), ginseng (Ren Shen), angelica sinensis (Dang Gui), tangerine peel (Chen Pi), cimicifuga foetida (Sheng Ma), bupleurum (Chai Hu), fried atractylodes (Chao Bai Zhu), and baked licorice (Zhi Gan Cao). The astragalus (Huang Qi) in this prescription could strengthen the center and benefit vital energy, raise yang and lift prolapsed zang-fu organs; Ginseng (Ren Shen), atractylodes (Bai Zhu), and glycyrrhiza (Gan Cao) could reinforce the spleen to replenish qi; tangerine peel (Chen Pi) could regulate the functional activities of qi; angelica sinensis (Dang Gui) could enrich the blood and invigorate the circulation of blood; cimicifuga foetida (Sheng Ma) and bupleurum (Chai Hu) could ascend up the spleen qi. The whole prescription could show an effect on reinforcing spleen and benefiting qi and lift prolapsed zang-fu organs by improving yang. The studies of modern pharmacology have confirmed that Buzhong Yiqi Decoction could improve the function of neuromuscular junction and the effect on improving myasthenia.^[26]^ It has been confirmed clinically that Buzhong Yiqi decoction combined with surgery has a reliable effect on the treatment of rectal prolapse. However, evidence from RCTs is inconsistent. With an increasing number of clinical trials, it is urgent to make a systematic evaluation of the efficacy of Buzhong Yiqi decoction in the treatment of rectal prolapse. In this study, we summarized the latest evidence on the efficacy of Buzhong Yiqi decoction combined with surgery in the treatment of rectal prolapse. The research also provided useful evidence to identify whether Buzhong Yiqi decoction was effective and safe for patients with rectal prolapse, which was beneficial to both clinical practice and health-related decision makers.

However, this systematic review had some limitations: there were differences in the doses and ingredients of the traditional Chinese medicine used in the included studies as well as differences in the surgical methods and the condition of the patient's disease. There may be some clinical heterogeneity. The course of disease was also different, which may have affected the results to some extent. Constrained by language ability, we only searched English and Chinese literature and may ignore studies or reports in other languages.

## Author contributions

**Data collection:** Yanpeng Xie and Yihua Fan.

**Funding support:** Xiaoen Chen and Xiangdong Yang.

**Literature retrieval:** Chen Yang and Renhong Wan.

**Software operating:** Yuanzhang Hu and Changyou Deng.

**Supervision:** Xiangdong Yang

**Writing – original draft:** Yanpeng Xie and Yihua Fan.

**Writing – review & editing:** Xiangdong Yang, Xiaoen Chen, and Xiangdong Yang.

## Abbreviations

Abbreviation: RCTs = randomized controlled trials.
